# Directionality of point mutation and 5-methylcytosine deamination rates in the chimpanzee genome

**DOI:** 10.1186/1471-2164-7-316

**Published:** 2006-12-13

**Authors:** Cizhong Jiang, Zhongming Zhao

**Affiliations:** 1 Virginia Institute for Psychiatric and Behavioral Genetics, Virginia Commonwealth University, P.O. Box 980126, Richmond, VA 23298-0126, USA; 2 Center for the Study of Biological Complexity, Virginia Commonwealth University, Richmond, VA 23284, USA

## Abstract

**Background:**

The pattern of point mutation is important for studying mutational mechanisms, genome evolution, and diseases. Previous studies of mutation direction were largely based on substitution data from a limited number of loci. To date, there is no genome-wide analysis of mutation direction or methylation-dependent transition rates in the chimpanzee or its categorized genomic regions.

**Results:**

In this study, we performed a detailed examination of mutation direction in the chimpanzee genome and its categorized genomic regions using 588,918 SNPs whose ancestral alleles could be inferred by mapping them to human genome sequences. The C→T (G→A) changes occurred most frequently in the chimpanzee genome. Each type of transition occurred approximately four times more frequently than each type of transversion. Notably, the frequency of C→T (G→A) was the highest in exons among the genomic categories regardless of whether we calculated directly, normalized with the nucleotide content, or removed the SNPs involved in the CpG effect. Moreover, the directionality of the point mutation in exons and CpG islands were opposite relative to their corresponding intergenic regions, indicating that different forces govern the nucleotide changes. Our analysis suggests that the GC content is not in equilibrium in the chimpanzee genome. Further quantitative analysis revealed that the 5-methylcytosine deamination rates at CpG sites were highly dependent on the local GC content and the lengths of SNP flanking sequences and varied among categorized genomic regions.

**Conclusion:**

We present the first mutational spectrum, estimated by three different approaches, in the chimpanzee genome. Our results provide detailed information on recent nucleotide changes and methylation-dependent transition rates in the chimpanzee genome after its split from the human. These results have important implications for understanding genome composition evolution, mechanisms of point mutation, and other genetic factors such as selection, biased codon usage, biased gene conversion, and recombination.

## Background

As the closest relative to the human, the chimpanzee has been one of the best model organisms for researchers from anthropologists to molecular biologists. The recent release of the chimpanzee genome sequences and its comparison with the human genome sequences shows that the two genomes differ only by about 35 million nucleotides, or 1.23% [1]. Knowing that the two genomes are so similar, what makes us human becomes the most interesting, yet challenging, question for biologists [2]. While it is important for us to investigate the pattern of these 35 million substitutions, especially those in the functional regions, the comparative analysis of the single nucleotide polymorphisms (SNPs) in both genomes should provide many more insights on how these two genomes have evolved at the nucleotide level and how these new mutations might contribute to the distinct human or chimpanzee traits, such as the ability of learning complex languages and habitual bipedality [3,4]. Note that most SNPs have been created relatively recently, i.e., less than 1 million years ago, compared to the divergence time of humans and chimpanzees, i.e., 5–7 million years ago [5-7].

Previous studies show that point mutations do not occur randomly and are dependent on sequence context [8-10]. The pattern of nucleotide changes such as G/C→A/T versus A/T→G/C in different sequence environments or genomic regions (e.g., intergenic regions and exons) could help us understand the compositional evolution in the genomes [11,12]. One recent analysis revealed a bias toward fixation of A/T→G/C mutations with no significant difference between the G/C→A/T and A/T→G/C changes at the polymorphic sites, suggesting that these two changes differed significantly between fixed and polymorphic sites in the 1.8-Mb noncoding regions examined [13]. Another prominent feature in the genome composition evolution is the hypermutability of methylated CpG dinucleotides. About 80% of the CpG dinucleotides are methylated at their 5 position on the cytosine ring in mammalian genomes; however, they often remain unmethylated in CpG islands, clusters of CpG dinucleotides in GC-rich regions [14]. The 5-methylcytosines (5^m^C) in CpG dinucleotides have a remarkably higher mutation rate (e.g., 10–50 times other transitional changes) of 5^m^C to T by deamination [15,16]. This mechanism, which is well known as the CpG effect, decreases the presence of CpG dinucleotides and GC content in vertebrate genomes [16,17]. While the CpG effect has been well documented, quantitative measurements could not be performed without the recent release of several mammalian genomes and their genome-wide polymorphism data [1,18,19]. One way to approach this is to measure the 5^m^C deamination rate, which is calculated by the difference between CpG transition rate and GpC transition rate [20], because the GpC dinucleotides are not methylated in mammalian genomes [21]. This analysis found that the 5^m^C deamination rates were highly dependent on local GC content in the human genome [20].

Early studies of mutation direction in mammalian genomes were limited to a small number of pseudogenes and functional regions [5,9,22-24]. Some recent studies of mutation patterns were mainly based on the substitution data between human and chimpanzee sequences [7,12,13,25]. While these studies provided abundant information of nucleotide changes in the human and chimpanzee genomes, some inconsistent results have been observed. For example, the rates of G/C→A/T and A/T→G/C mutations varied in different regions, leading to debate on the GC content equilibrium in the genomes [13,22,26,27]. Importantly, the results from these studies were based on the data in specific regions; therefore, they may not represent the whole chimpanzee genome. The recent release of more than 1 million chimpanzee SNPs provides an alternative way to systematically examine the mutation pattern in the chimpanzee genome, in particular, to compare the features in the categorized genomic regions. To our knowledge, there is no genome-wide analysis of mutation direction and CpG effects in the chimpanzee genome.

In this study, we performed a comprehensive analysis of the mutation pattern in the chimpanzee genome using the SNP data publicly available in the National Center for Biotechnology Information (NCBI) dbSNP database. We inferred the ancestral information of these chimpanzee SNPs by mapping them to human genome sequences and then used it to estimate the mutational spectra in the overall genome and in the categorized genomic regions. We further compared the directionality of the nucleotide changes by normalization with the GC content in the regions where the SNPs occurred or by removal of the SNPs involved in the CpG effect. Finally, to quantitatively examine the CpG effect in the chimpanzee genome, we estimated and compared the 5^m^C deamination rates in the chimpanzee genome and genomic regions. Our results, especially in the exons and CpG islands, revealed many important features in the chimpanzee genome, or more broadly, in mammalian genomes.

## Results

### Mutation direction in the chimpanzee genome

We identified 702,590 biallelic SNPs that were uniquely mapped in the non-repetitive sequences in the chimpanzee genome and had at least 100 nucleotides at each flanking side of the SNPs. We used this SNP dataset to infer the mutation direction in the chimpanzee genome. There were 588,918 (84%) SNPs that could be reliably mapped in the human genome, thus, their ancestral alleles were inferred (see Methods). The first row in Table 1 shows the frequencies of nucleotide changes in the chimpanzee genome. These frequencies varied greatly among the different types of nucleotide changes, indicating the non-randomness of the nucleotide changes in the chimpanzee genome. The frequency of each type of transitional mutation (A→G, T→C, G→A, and C→T) was approximately four times that of transversional mutation (e.g., A→C). As expected, the frequencies for each pair of nucleotide changes, such as A→G and T→C, were nearly the same, reflecting complementary DNA strand symmetry. Throughout the rest of this paper, we will use A:T→G:C to denote such a pair of nucleotide changes. In addition, we will use A/T→C/G to denote the nucleotide changes from A or T to C or G.

Importantly, considering the large number of genome-wide SNPs analyzed (Table 1), the frequency of C→T (17.7%) is notably higher than that of T→C (16.0%, χ^2 ^test, *P *= 2.5 × 10^-114^) and the frequency of G→A (17.6%) is notably higher than that of A→G (16.1%, χ^2 ^test, *P *= 7.5 × 10^-85^). This might be partially caused by the hypermutability of the methylated CpG (^m^CpG) dinucleotides, which lead to TpG by deamination [17,28]. This feature was consistently observed when we examined the mutation direction in the categorized genomic regions (Table 1). In fact, the difference became much larger in the exons and CpG islands, which had higher GC content than the overall genome. For example, for exons with GC content of 51.7% compared to the genome average of 40.0%, the frequency difference between C→T and T→C was 17.0% (28.3 – 11.3%) compared to 1.7% (17.7 – 16.0%) in the genome. Upon further examination in the intergenic regions with different GC content bins, this feature is highly dependent on the GC content (See Additional file 1).

The non-randomness of nucleotide changes observed in the overall genome was consistently observed in the categorized genomic regions. As expected, mutational spectra were similar in intergenic regions and in introns because both regions had similar GC content and are considered to be (nearly) selectively neutral. Interestingly, although both the exons and CpG islands had higher GC content than the genome average, their mutation direction differed. For each type of transitional mutation, its frequency in the exons was higher than that in the CpG islands, and conversely, for each type of transversional mutation, its frequency in the exons was lower than that in the CpG islands. It is worth noting that the frequencies of G:C→A:T in the exons were the highest among all genomic regions, even though the GC content in the exons was lower than that in the CpG islands (Table 1). Finally, the changes from G or C to any other nucleotide dominated in the CpG islands. For example, the nucleotide changes G:C→T:A and G:C→C:G had the highest frequencies among the categories. These results reflect the influence of high GC content and, in general, a lack of the CpG effect in CpG islands [14,29].

Intergenic regions are usually considered to be selectively neutral, thus, are suitable for studying the pattern of spontaneous point mutation [6]. Therefore, we examined the frequencies of nucleotide changes in intergenic regions grouped by different GC content bins. The frequencies of nucleotide changes in each GC content bin are shown in Additional file 1. In summary, the frequencies of changes from G or C to any other nucleotide increased when the GC content increased. Linear regression analysis indicates a significant correlation between the frequency of each type of nucleotide change and the GC content (See Additional file 2).

### Normalized frequencies of nucleotide changes in the chimpanzee genome

The results presented above indicate that the frequencies of nucleotide changes depend on the GC content, or nucleotide compositions, in the sequences examined. They represent the observed frequencies of recent nucleotide changes in the chimpanzee genome. Because nucleotide compositions vary across the genome and among the genomic regions, we estimated the relative mutation frequencies by normalizing the nucleotide changes with their nucleotide content in the sequences (see Methods). The normalized frequencies represent the expected nucleotide changes in a random sequence, where each nucleotide is found in equal frequency (i.e., 25%) [9].

Table 2 shows the normalized frequencies of nucleotide changes in the chimpanzee genome and in the genomic regions. Overall, after the normalization, the nucleotide changes from A or T to G or C decreased, while the nucleotide changes from G or C to A or T increased in the genome or genomic categories that had < 50% GC content (genome, intergenic regions, genes, and introns). The opposite adjustments were shown in the genomic categories that had > 50% GC content (exons and CpG islands). Strikingly, in the chimpanzee genome, the difference of the frequencies of C→T and T→C changes increased from 1.7% (17.7 – 16.0%, Table 1) before the normalization to 8.4% (21.1 – 12.7%, Table 2) after the normalization. In the CpG islands the normalized frequencies became close to those in the overall genome, this contrasts to the large difference observed before the normalization. However, large differences were still observed in the exons from the overall genome. For example, the frequency of C→T was 27.7% in the exons compared to the 21.1 % in the genome (Table 2).

### Mutation direction after excluding the CpG effects

The mutational spectra discussed above were strongly affected by the hypermutability of CpG dinucleotides in the genome [30]. To examine the mutational spectrum without such effects in the chimpanzee genome, we excluded the nucleotide changes CpG→TpG/CpA. Note that CpG→TpG/CpA changes might result from the deamination events at ^m^CpGs or spontaneous mutations at CpGs. The results after normalization with nucleotide content are shown in Table 3. The frequencies of mutations G:C→A:T decreased dramatically among all categories. This strong decrease helped the frequencies of all other mutation types to increase (Table 3). The extent of the frequency decrease for G:C→A:T was the strongest in exons: 19.2% (from 54.4% to 35.2%). This is nearly twice that in the intergenic or intronic regions (Table 4). Furthermore, when we compared the exons with the intergenic regions that had similar GC content (50–55%), the frequency decrease in the exons was stronger than that (12.8%) in the corresponding intergenic regions. In contrast, the frequency decrease of G:C→A:T in the CpG islands was 11.2%, weaker than that (16.0%) in the intergenic regions that had similar GC content (55–75%). Assuming no CpG effect in CpG islands, these comparative results indicate that the influence of CpG→TpG/CpA mutations was strong in intergenic regions and even stronger in exons.

Table 4 also shows that when the GC content in the intergenic regions increased, the frequency decrease of G:C→A:T became stronger. For example, the frequency decrease was 7.1% in the GC content bin < 35%; this compared with the 17.4% in the GC content bin ≥ 60%. Linear regression analysis indicate that the correlation between the frequency decrease and GC content was significant (R^2 ^= 0.99, *p *< 0.0001).

### 5^m^C deamination rates

**Figure 1:**
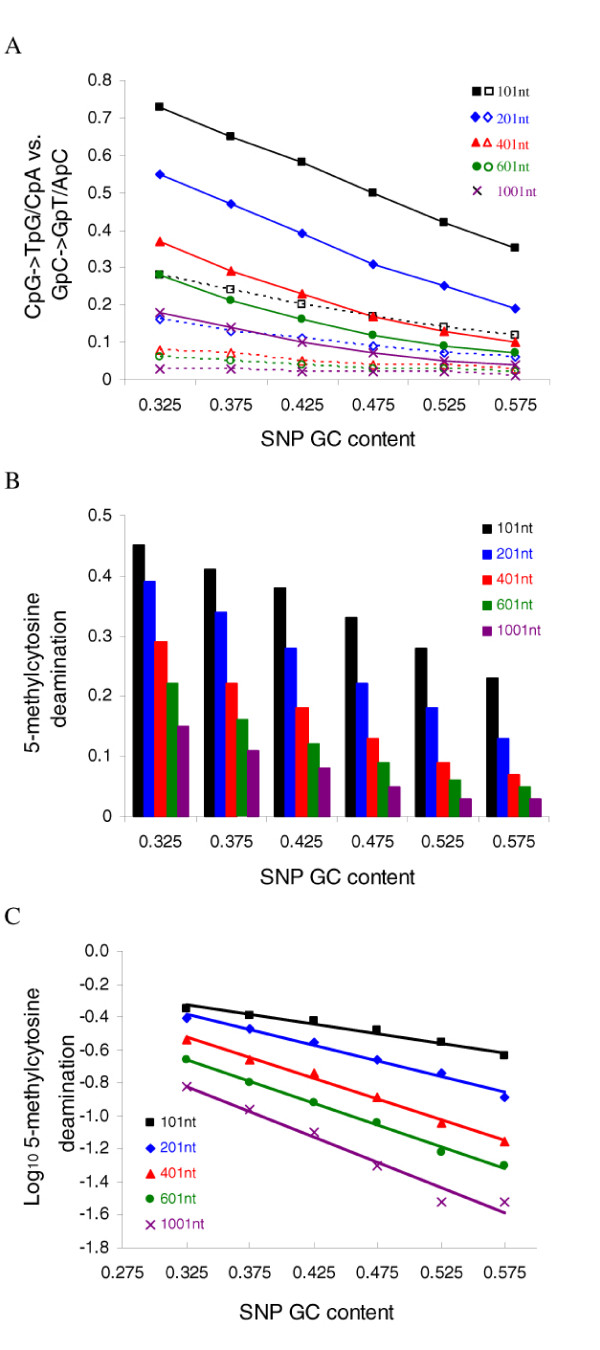
**Rates of CpG transition, GpC transition, and 5^m^C deamination varied with local GC content and SNP sequence length**. (A) Rates of CpG→TpG/CpA per CpG dinucleotide (solid line) and GpC→GpT/ApC per GpC dinucleotide (dashed line) in the SNP flanking sequences. SNP GC content was calculated from the SNP flanking sequences. (B) 5^m^C deamination rates, measured by the difference between the rates of CpG transition and GpC transition in A. (C) Plot of log_10_(5^m^C deamination rate) versus SNP GC content.

The results presented above consistently indicate the influence of the hypermutability of methylated CpGs on the mutational spectrum in the chimpanzee genome, especially in the exonic regions. We extended our analysis to examine the 5^m^C deamination rate, which could be measured by the difference between the CpG transition rate and GpC transition rate [20]. This analysis is based on the fact that GpC dinucleotides are not methylated in mammalian genomes [21] and the assumption that the mutation rate of the unmethylated CpG to TpG is equal to the rate of GpC to GpT. We collected 94,755 transition mutations that occurred in ancestral CpG or GpC dinucleotides. Then we calculated the CpG transition rate by the number of CpG→TpG/CpA per CpG dinucleotide in the SNP flanking sequences and GpC transition rate by the number of GpC→GpT/ApC per GpC dinucleotide in the SNP flanking sequences. To examine the dependence of the rates on GC content we grouped these SNPs according to the GC content in their flanking sequences. Figure 1A shows that the CpG transition rates were remarkably higher than the corresponding GpC transition rates. This difference became larger when the length of the SNP flanking sequences decreased, or when the GC content was smaller (Figure 1B). We next used the method in Fryxell and Moon [20] to plot log_10_(5^m^C deamination rate) versus GC content for each length category. Figure 1C shows the linear relationship between the log_10_(5^m^C deamination rate) and the local GC content of SNPs. The slope values were -1.2, -1.9, -2.5, -2.8, and -3.1, respectively, for the length of 101, 201, 401, 601, and 1001 nt.

**Figure 2:**
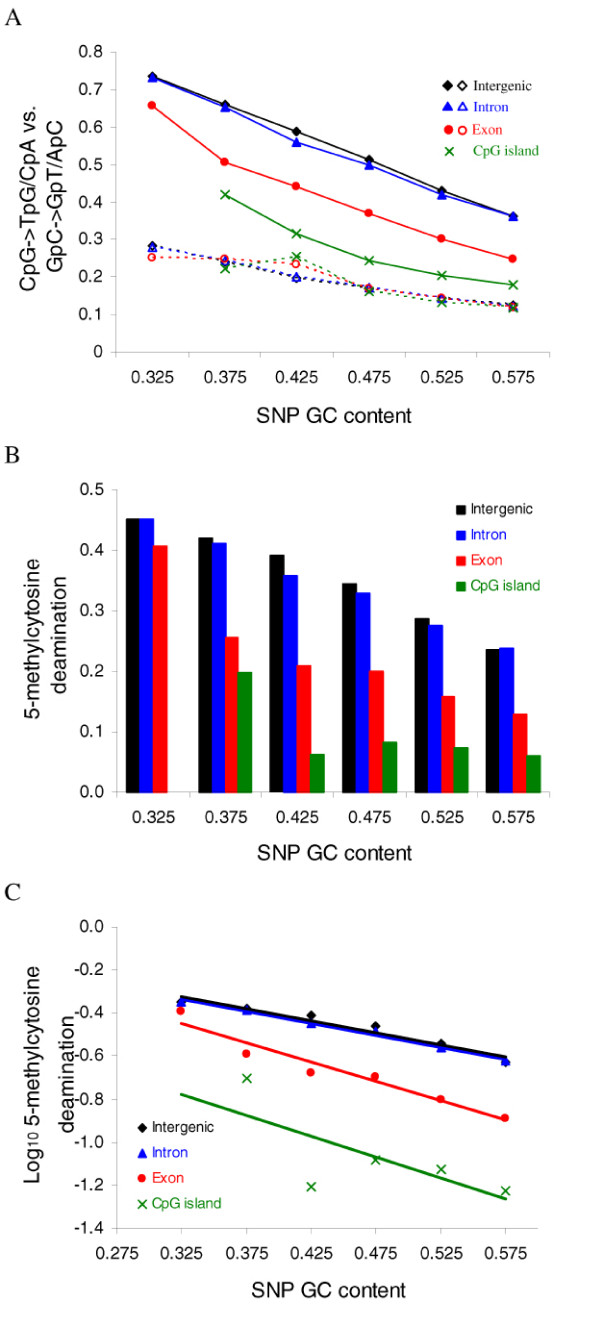
**Rates of CpG transition, GpC transition, and 5^m^C deamination varied with local GC content and among the genomic regions**. (A) Rates of CpG→TpG/CpA per CpG dinucleotide (solid line) and GpC→GpT/ApC per GpC dinucleotide (dashed line) in the SNP flanking sequences (length category 101 nt). In CpG islands, the rates at GC content bin 0.325 were not calculated due to the insufficient number of SNPs. (B) 5^m^C deamination rates, measured by the difference between the rates of CpG transition and GpC transition in A. (C) Plot of log_10_(5^m^C deamination rate) versus SNP GC content.

We next examined the CpG transition rates and GpC transition rates in the intergenic regions, introns, exons, and CpG islands. Figure 2A shows how in each GC content bin the CpG transition rates varied among these genomic regions, though the GpC transition rates were nearly the same. Again, the linear relationship was observed in all genomic categories in their plots of log_10_(5^m^C deamination rate) over GC content. The slope values were -1.1 (intergenic regions), -1.1 (introns), -1.8 (exons), and -1.9 (CpG islands), respectively.

**Figure 3:**
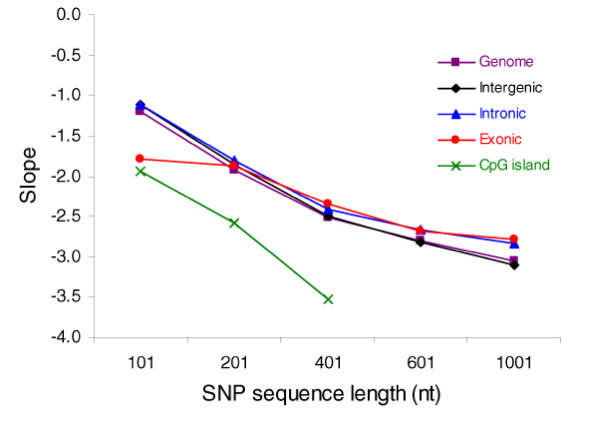
**Slopes of linear regression lines**. In CpG islands, the slopes for lengths 601 and 1001 nt were not included due to the insufficient number of SNPs in low GC-content bins.

In Figure 2, the GC content was calculated in the SNP length category 101 nt. We did similar analysis in the genomic regions for other SNP length categories. While the strong dependence of GC content was consistently observed in these analyses, the slope values varied (Figure 3). Notably, the slope values for the exons at length categories of 201, 401, 601, and 1001 were close to that for the genome, intergenic regions, or introns (Figure 3). This is likely due to the inclusion of sequences from the neighboring regions (e.g., introns) because of the short length of exons. While there is no report of the average length of exons in the chimpanzee genome, the average size of exons was reported to be 145 bp in the human genome [16]. Finally, the slopes in absolute value in the CpG islands were much greater than those in other genomic categories.

## Discussion

In this study, we examined the mutation direction in the chimpanzee genome and its genomic regions by three approaches: calculation of the frequency for each type of nucleotide change, normalization by nucleotide content, and removal of the CpG→TpG/CpA SNPs. The mutational spectra by these three approaches represent, respectively, the observed sequence mutability, relative sequence mutability, and sequence mutability without the CpG effect in the chimpanzee genome. Our results indicate that nucleotide changes were not random in the chimpanzee genome and varied greatly among the categorized genomic regions. This is in contrast to the random mutation model that each nucleotide changes to any other with the same frequency [31]. In the overall genome, the normalized frequency of G/C→A/T was 51.7%, higher than that (32.3%) of A/T→G/C. This suggests that point mutation has a trend towards increasing the AT content in the chimpanzee genome. This trend was further observed in all genomic categories before or after normalization (Tables 1 and 2) and is consistent with previous finding that the GC content in the human genome is decreasing due to a uniform mutational preference for A:T pairs [32]. We further examined whether the GC content in the chimpanzee genome has undergone statistical equilibrium. Given no natural selection and independence of point mutation, according to the Sueoka (1962) equation [33], the GC content at equilibrium (*c*) is equal to *v*/(*u*+*v*) where *u *and *v *are the rates of G/C→A/T and A/T→G/C, respectively. We found that the GC content in the overall genome, intergenic regions and introns appeared nearly at equilibrium; however, our further analysis in the intergenic regions with different GC content indicated that such an overall observation was too simple. The intergenic regions with low GC content (e.g., <35%) are expected to have more G/C→A/T changes, in contrast, the intergenic region with high GC content (e.g., ≥ 60%) are expected to have more A/T→G/C changes (See Additional file 1).

Early studies have shown that point mutation is correlated with GC content [13,22]. To remove the influence of GC content, we compared the mutational spectrum in exons with the intergenic regions that had similar GC content (50–55%). The directionalities of the point mutation in these two categories were different. The frequency of G:C→A:T was much higher in the exons (55.6%) than that in the intergenic regions (47.3%). Conversely, the frequency of A:T→G:C in the exons (23.0%) was nearly the same as that (22.7%) in the intergenic regions (Table 1, Additional file 1). After removal of CpG→TpG/CpA SNPs and normalization with nucleotide content, the frequency decrease of G:C→A:T in the exons (19.2%) was stronger than that (12.8%) in the intergenic regions with GC content 50–55% (Table 4). These comparative results indicate that, even after excluding the factor of GC content, G:C→A:T and CpG→TpG/CpA mutations occurred more frequently in exons than in intergenic regions, suggesting stronger CpG effects in exons. These results support the previous finding of that the faster synonymous rate in exons than in noncoding DNA sequences is largely due to the overabundance of synonymous sites involved in CpG dinucleotides [34]. To further investigate other genetic factors in exons, we compared the mutation direction at the fourfold degenerate (FFD) sites, which are largely free from selection, and the non-FFD sites, which are often under selection [31,34,35]. We identified 1296 FFD SNPs and 3120 non-FFD SNPs. The frequency of G:C→A:T at the FFD sites (59.9%) was much higher than that (53.8%) at the non-FFD sites. After normalization, the frequency of G:C→A:T was 56.4% for FFD SNPs and 53.3% for non-FFD SNPs. Further, after removal of CpG→TpG/CpA SNPs and normalization with nucleotide content, the frequency of G:C→A:T at the FFD sites (33.2%) became lower than that (35.8%) at the non-FFD sites; this led to a stronger frequency decrease of G:C→A:T at the FFD sites (23.2%) than at the non-FFD sites (17.6%). These results provide further support of the overabundance of synonymous sites in CpGs [34]. They also suggest that mutation direction has been influenced by selection and biased codon usage. However, the effects of selection and codon usage seem moderate because the frequency difference between exons and intergenic regions was even larger. Overall, our analysis suggests that directionality of point mutations in exons was moderately influenced by selection and codon usage but mainly caused by the GC content and mutational forces (e.g., hypermutability at CpG sites).

Interestingly, when we compared the results in the CpG islands with those in the intergenic regions with similar GC content (55–75%), we found the opposite pattern from the exons. The frequency of G:C→A:T in the CpG islands was 46.0%, less than the 53.8% in the intergenic regions (Table 1, Additional file 1). After the removal of CpG→TpG/CpA SNPs and normalization with nucleotide content, the frequency decrease of G:C→A:T was 11.2% in the CpG islands, weaker than the 16.0% in the intergenic regions (Table 4). Our further analysis revealed that 66.8% of the ancestral CpG dinucleotides where SNPs located were mutated to TpGs/CpAs in the CpG islands; this compared with the 86.6% in the intergenic regions with GC content 55–75%, or 91.6% in the overall intergenic regions, 91.9% in introns, 90.2% in exons, and 91.6% in the overall genome. These comparisons provide strong evidence of the absence of CpG effects in the chimpanzee CpG islands.

CpG dinucleotides mutate at a high rate because the methylated cytosines change to thymidines by deamination. The mutation rate of ^m^CpG to TpG was estimated to be 10–50 folds higher than other transitions [17,36]. Our study revealed that the deamination rates were exponentially correlated with the local GC content of the SNPs, or the log_10_(5^m^C deamination rate) was linearly correlated with the local GC content (Fig. 1C). This is consistent with a previous study in humans by Fryxell and Moon [20]. However, our observations are different from their other conclusions, which state that the slopes in the linear regression analysis of log_10_(5^m^C deamination rate) versus GC content were the same regardless of the lengths over which GC content was calculated or the genomic regions where the SNPs located [20]. In our study, the slopes decreased from -1.2 to -3.1 when the lengths of the SNP flanking sequences increased from 101 to 1001 nt (Fig. 3). Further, the slope values in the CpG islands and exons were different from those in the intergenic and intronic regions (Fig. 3). The reasons that we had similar slope values in the intergenic regions and introns are that the sequences had the similar nucleotide composition (e.g., GC content) and both regions are generally considered to be selectively neutral. Overall, our observations should be accurate because the SNPs are strongly biased on their local sequences and CpG effects depend on the genomic regions as well as the GC content of the sequences [34,37].

In this study, we analyzed the frequencies of nucleotide changes and mutation rates in the chimpanzee genome and categorized genomic regions. The observed results were influenced by many genetic factors such as the mutation rate, recombination rate, gene conversion, and biased DNA mismatch repair. One recent analysis of a substitution pattern in 14.3 Mb of primate noncoding regions revealed the positive correlation between the recombination rate and GC content, suggesting that recombination drives the evolution of base composition in genomes [12]. The mutation pattern might also be influenced by biased gene conversion. Galtier et al [11] proposed that gene conversion from a recombination event may be repaired with a bias toward G:C pairs. In our study, both CpG and GpC transition rates decreased when GC content increased (Figure 1A), thus, our results can not rule out the possible effect of biased gene conversion. Further investigations, i.e., separating SNPs into different recombination rates, shall help uncover the effects of these genetic factors on mutation pattern.

To compare the pattern observed in the chimpanzee genome, we performed a similar analysis using the SNPs in the human genome. We found that the frequency of each type of point mutation in chimpanzees was generally close to that in humans, indicating the similar mutation pattern in these two closely related genomes (See Additional file 3). For any type of transversion, the frequency difference in the two genomes is less than 0.5% in any genomic category. For A→G or T→C mutations, humans had ~0.5–1.0% more frequency than chimpanzees in all genomic categories except for CpG islands. Conversely, for G→A or C→T mutations, chimpanzees generally had a higher frequency compared with humans. While these differences were small, there is one exception. The frequency of C→T in the chimpanzee exons was 28.3%, ~2.8% higher than that (25.5%) in the human exons. This large difference might be partially attributed to the small number of SNPs in exons since the frequencies of each complementary pair (e.g., C→T and G→A) were not nearly the same in exons (Table 1). It is also possible that the G:C→A:T mutations in the chimpanzee exons occurred more frequently compared to the human exons. Further examination is needed to understand this large difference. Moreover, we compared the frequency difference for each type of mutation in each syntenic chromosome pair between the chimpanzee and human (See Additional file 4). The differences above were similarly observed in most of the chromosome pairs. Finally, we examined the deamination rates in the human genome and found that the rates were highly dependent on the local GC content and lengths of SNP flanking sequences over which the local GC content was calculated, and varied among the different genomic categories (unpublished data).

Analyses restricted to genome databases have potential biases. In this study, potential biases could come from errors in the SNP and genome data, insufficiency of data, and incorrect inference of mutation direction. First, most of the SNPs used in this study were discovered by a systematic comparison of the sequences from eight lineages: the primary donor (Clint), four other western African chimpanzees, and three central African chimpanzees from the Chimpanzee Sequencing and Analysis Consortium [1]. The quality of these SNPs seems high. Among the 704,687 SNPs that were biallelic and uniquely mapped in the non-repetitive chimpanzee sequences, nearly 100% (702,590) had a minimum of 200 nucleotides in the flanking sequences. Among these 702,590 SNPs, 84% (588,918) could be uniquely mapped in the human genome even though stringent criteria were applied for SNP mapping (see Methods). Moreover, the quality of both the human and chimpanzee genome reference sequences seem high. The human genome reference sequence (build 35) has only 341 gaps in the 2.85 billion nucleotides, covers ~99% of the euchromatic genome, and has an error rate of only ~1 event per 100,000 bases [38]. The chimpanzee assembly (build 1) covers ~94% of the chimpanzee genome with >98% of the sequence in high-quality bases (i.e., error rate is ≤ 10^-4^) [1]. Since only those SNPs that were biallelic and uniquely mapped in the non-repetitive chimpanzee and human sequences were used in this study, the artifacts, if any, should have limited effect on our results. Second, the number of SNPs seems sufficient to draw reliable conclusions. In the estimation of mutational spectrum, the frequencies in a pair of nucleotide changes (e.g., G→A and C→T) were close in most of the categories we investigated (Table 1). In our estimation of 5^m^C deamination rates, the number of SNPs in some of the GC content bins in the CpG islands was not sufficient; however, this has little effect on the conclusions. Finally, the mutation direction was inferred by comparing the two alleles of a chimpanzee SNP with its mapped allele in the human genome. This is based on the low point mutation rate in both genomes and a short divergence of time between the human and chimpanzee. However, the transition rate at the methylated CpG dinucleotides is ~10–50 times higher than at other sites [17,36]. An opposite mutation direction might be inferred for a [C/T]G chimpanzee SNP with its ancestral sequence being CG and human sequence being TG [27]. In this study, we used stringent criteria to determine whether a chimpanzee SNP is mapped to the human genome (see Methods). Among the 588,918 chimpanzee SNPs we analyzed, 4852 were also polymorphic in the human genome, including only 442 [C/T]G SNPs whose human sequences were TGs and 392 C [A/G] SNPs whose human sequences were CAs. Given that a portion of them might be incorrectly inferred, the errors should be minimal in our estimation of mutation direction.

## Conclusion

We performed a detailed examination of mutation direction using 588,918 SNPs that were uniquely mapped in the non-repetitive chimpanzee sequences and whose ancestral alleles could be inferred by mapping them to the human genome reference sequences. The directionalities of these SNPs were compared among the different genomic regions, by normalization with the nucleotide content, and by removal of the CpG→TpG/CpA SNPs. Overall, point mutation occurred non-randomly, was dependent on GC content, and varied among the categorized genomic regions. Importantly, the directionality of point mutation in exons and CpG islands showed opposite patterns relative to their corresponding intergenic regions. Our analysis provides evidence of strong CpG effects in the chimpanzee genome but not in the CpG islands. Further quantitative analysis revealed that the 5^m^C deamination rates were exponentially dependent on the local GC content and varied with the lengths of local SNP sequences and among the categorized genomic regions.

## Methods

### Chimpanzee SNP and genome sequence data

We downloaded 1,542,718 reference SNPs in the chimpanzee genome and their annotations from the NCBI dbSNP database (build 125, released on October 25, 2005) [39]. We wrote a Perl script to extract those SNPs that were biallelic and uniquely mapped in the chimpanzee genome. This process extracted a total of 1,432,682 (92.9%) SNPs.

We downloaded the assembled chimpanzee chromosomal sequences from the NCBI (build 1, released on November 23, 2004) [40]. In addition, we downloaded two files: 'masking_coordinates.gz', which provided locations of all repetitive sequences in the genomic contigs, and 'seq_contig.md', which provided locations and orientations of the genomic contigs in the assembled chromosomal sequences. The non-repetitive sequences and their locations in the chimpanzee genome were obtained according to these files. Next, we identified SNPs in the non-repetitive sequences by comparing the locations of SNPs and non-repetitive sequences in the assembled chromosomes. This procedure resulted in 704,687 SNPs, among them, 702,590 had a minimum of 100 nucleotides in each flanking sequence of SNPs. These SNPs were formatted to have 100 nucleotides at each side and used in this study.

### Categorization of SNPs into genomic regions

We downloaded the chimpanzee genes and their annotations from the Ensembl database (v35, released in March 2005) [41]. We wrote another Perl script to retrieve the positions of genes and exons on the chromosomes. To obtain high-quality data for our study, we applied the following high-stringent criteria to identify genes, exons, introns, intergenic regions, and CpG islands. (1) For genes and exons, we only selected those annotated as "known" genes. (2) For introns, we selected those annotated as introns in the known and predicted genes but excluded those introns that might be also annotated as exons in the alternative transcripts. (3) For intergenic regions, we selected those sequences without overlap with any known or predicted genes. (4) CpG islands in the chimpanzee genome were identified by the CpG island searcher program CpGi130 [42], using the stringent search criteria of GC content ≥ 55%, Obs_CpG_/Exp_CpG _≥ 0.65, and length ≥ 500 bp [43]. Next, we identified SNPs in these genomic regions by comparing their locations in the assembled chromosomes. This procedure resulted in 452,671, 194,579, 100,038, 4897, and 8910 SNPs in intergenic regions, genes, introns, exons, and CpG islands, respectively. Note that the combined number of SNPs in introns and exons is less than the number of SNPs in genes due to our selection criteria.

To examine whether the mutation directions are correlated with GC content, we subcategorized the non-repetitive intergenic regions into different GC content bins. The GC content in the non-repetitive intergenic sequences was calculated using a scanning window size of 500 bp. The GC content bins included ≤ 0.35, 0.35 – 0.40, 0.40 – 0.45, 0.45 – 0.50, 0.50 – 0.55, 0.55 – 0.60, and ≥ 0.60. Correspondingly, intergenic SNPs in each GC content bin were identified by comparing their positions in the assembled chromosomes.

### Inference of mutation direction

We inferred the mutation direction of chimpanzee SNPs by comparing their outgroup (i.e., ancestral) sequences in the human genome. Because the chimpanzee and human genomes are highly similar (i.e., ~99%) [44] and the length of the SNP sequences is 201 bp (including the polymorphic site and 100 bp at each side, see above), we used the Megablast program (version 2.2.11) [45] to map chimpanzee SNPs to the human genome. We downloaded the assembled human genome sequences from the NCBI (build 35, released on August 26, 2004) [40] and masked the repetitive sequences. We ran Megablast program by taking an E-value of -80 and X-drop-off value of 180. Then, we wrote a Perl script to parse the Megablast output. A chimpanzee SNP was mapped to the human genome when the high-scoring segment pair (HSP) satisfied the following criteria: (1) the identity score was ≥ 95%; (2) the length of the alignment was in a range of 196–206 bp; (3) the polymorphic site was located in the middle (positions 96–106) of the alignment; (4) the immediate adjacent 5 nucleotides of the polymorphic site at each side were identical; (5) the mapped human allele of the chimpanzee SNP was one of the two alleles of that SNP; and (6) only one HSP satisfied the above criteria. We tested 702,590 SNPs and found that 588,918 (83.8%) met the above criteria. The mutation direction of these SNPs was inferred by comparing the alleles of chimpanzee SNPs and their corresponding (ancestral) human nucleotides. For example, if one SNP has two polymorphic alleles C and T and the mapped human allele is C, the mutation direction would be inferred to be C→T.

### Frequencies of nucleotide changes and normalized frequencies of nucleotide changes

The frequency of each nucleotide change was calculated by

fi−>j=ni−>j∑i∑j≠ini−>j×100%
 MathType@MTEF@5@5@+=feaafiart1ev1aaatCvAUfKttLearuWrP9MDH5MBPbIqV92AaeXatLxBI9gBaebbnrfifHhDYfgasaacH8akY=wiFfYdH8Gipec8Eeeu0xXdbba9frFj0=OqFfea0dXdd9vqai=hGuQ8kuc9pgc9s8qqaq=dirpe0xb9q8qiLsFr0=vr0=vr0dc8meaabaqaciaacaGaaeqabaqabeGadaaakeaacqWGMbGzdaWgaaWcbaGaemyAaKMaeyOeI0IaeyOpa4JaemOAaOgabeaakiabg2da9maalaaabaGaemOBa42aaSbaaSqaaiabdMgaPjabgkHiTiabg6da+iabdQgaQbqabaaakeaadaaeqbqaamaaqafabaGaemOBa42aaSbaaSqaaiabdMgaPjabgkHiTiabg6da+iabdQgaQbqabaaabaGaemOAaOMaeyiyIKRaemyAaKgabeqdcqGHris5aaWcbaGaemyAaKgabeqdcqGHris5aaaakiabgEna0kabigdaXiabicdaWiabicdaWiabcwcaLaaa@5054@

where *n*_*i *-> *j *_is the counts of nucleotide changes from the *i*-th type to the *j*-th type (*i*, *j *= A, C, G or T).

In a random sequence, the frequency of each nucleotide change was normalized by

fi−>j=ni−>j/Ni∑i∑j≠i(ni−>j/Ni)×100%
 MathType@MTEF@5@5@+=feaafiart1ev1aaatCvAUfKttLearuWrP9MDH5MBPbIqV92AaeXatLxBI9gBaebbnrfifHhDYfgasaacH8akY=wiFfYdH8Gipec8Eeeu0xXdbba9frFj0=OqFfea0dXdd9vqai=hGuQ8kuc9pgc9s8qqaq=dirpe0xb9q8qiLsFr0=vr0=vr0dc8meaabaqaciaacaGaaeqabaqabeGadaaakeaacqWGMbGzdaWgaaWcbaGaemyAaKMaeyOeI0IaeyOpa4JaemOAaOgabeaakiabg2da9maalaaabaGaemOBa42aaSbaaSqaaiabdMgaPjabgkHiTiabg6da+iabdQgaQbqabaGccqGGVaWlcqWGobGtdaWgaaWcbaGaemyAaKgabeaaaOqaamaaqafabaWaaabuaeaacqGGOaakcqWGUbGBdaWgaaWcbaGaemyAaKMaeyOeI0IaeyOpa4JaemOAaOgabeaakiabc+caViabd6eaonaaBaaaleaacqWGPbqAaeqaaOGaeiykaKcaleaacqWGQbGAcqGHGjsUcqWGPbqAaeqaniabggHiLdaaleaacqWGPbqAaeqaniabggHiLdaaaOGaey41aqRaeGymaeJaeGimaaJaeGimaaJaeiyjaucaaa@5953@

where *n*_*i *-> *j *_is the counts of nucleotide changes from the *i*-th type to the *j*-th type (*i*, *j *= A, C, G or T) and *N*_*i *_is the total counts of nucleotide *i *in the sequences.

### Transition rate in CpG and GpC dinucleotides

Among the 588,918 SNPs that satisfied the mapping criteria, 94,755 were transition mutations that occurred in ancestral CpG or GpC dinucleotides. They were further categorized into intergenic regions, introns, exons, and CpG islands. We identified 57,642 transition mutations in intergenic regions, 13,661 in introns, 1611 in exons, and 1861 in CpG islands. For each of these transition SNPs, we obtained 500 nucleotides at each flanking side based on the flanking sequences and the mapped contig sequences [30].

In the flanking sequences of these SNPs, we counted the CpG and GpC dinucleotides and calculated the GC content. We used SNP sequences whose lengths were 101, 201, 401, 601, and 1001 nucleotides, respectively. Next, we calculated CpG transition rate by the number of CpG→TpG/CpA per CpG dinucleotide and GpC transition rate by the number of GpC→GpT/ApC per GpC dinucleotide.

## Authors' contributions

CJ participated in the data preparation, carried out data analysis, and helped to draft the manuscript. ZZ conceived of the study, participated in its design and analysis, and helped to draft the manuscript. All authors read and approved the final manuscript.

## Supplementary Material

Additional file 1 Frequencies of nucleotide changes in intergenic regions. Supplementary Table S1 – Frequencies (%) of nucleotide changes in intergenic regions. Click here for file

Additional file 2 Linear regression of the frequency of nucleotide changes versus GC content in intergenic regions. Supplementary Figure S1 – Linear regression of the frequency of each type of nucleotide changes versus GC content in intergenic regions. Click here for file

Additional file 3 Frequency difference of nucleotide changes between chimpanzees and humans in each genomic category. Supplementary Figure S2 – Frequency difference of nucleotide changes between chimpanzees and humans in each genomic category. Click here for file

Additional file 4 Frequency difference of nucleotide changes for each pair of syntenic chimpanzee and human chromosomes. Supplementary Figure S3 – Frequency difference of nucleotide changes for each pair of syntenic chimpanzee and human chromosomes. Click here for file

## Figures and Tables

**Table 1:** Frequencies (%) of nucleotide changes in the chimpanzee genome and the categorized genomic regions

Category	GC%^a^	No. of SNPs	A→G	T→C	G→A	C→T	A→C	T→G	G→T	C→A	A→T	T→A	G→C	C→G
Genome	40.0	588,918	16.1	16.0	17.6	17.7	4.3	4.3	4.1	4.1	3.6	3.5	4.4	4.4
Intergenic regions	39.4	376,235	16.2	16.2	17.1	17.2	4.4	4.3	4.2	4.2	3.7	3.7	4.4	4.4
Genes	41.0	164,294	15.9	15.8	18.3	18.6	4.2	4.2	3.7	3.8	3.3	3.3	4.5	4.4
Introns	39.7	84,542	16.3	15.9	17.7	18.0	4.4	4.3	3.7	3.8	3.4	3.4	4.7	4.4
Exons	51.7	4416	11.7	11.3	27.3	28.3	2.2	2.2	2.9	3.0	1.5	1.4	4.0	4.2
CpG islands	62.0	7205	8.7	9.0	23.1	22.9	2.7	3.1	5.9	6.0	1.9	1.9	7.4	7.4

^a^GC content was calculated in the non-repetitive sequences in each genomic category except in the CpG islands.

**Table 2:** Normalized frequencies (%) of nucleotide changes in the chimpanzee genome and the categorized genomic regions

Category	A→G	T→C	G→A	C→T	A→C	T→G	G→T	C→A	A→T	T→A	G→C	C→G
Genome	12.8	12.7	20.9	21.1	3.4	3.4	4.8	4.9	2.8	2.8	5.3	5.2
Intergenic regions	12.7	12.6	20.6	20.7	3.4	3.4	5.1	5.1	2.9	2.9	5.3	5.3
Genes	12.9	12.8	21.4	21.6	3.4	3.4	4.3	4.5	2.7	2.6	5.3	5.2
Introns	12.8	12.5	21.2	21.5	3.5	3.4	4.4	4.6	2.7	2.7	5.6	5.3
Exons	12.3	11.8	26.7	27.7	2.3	2.3	2.9	3.0	1.6	1.4	4.0	4.1
CpG islands	12.1	12.6	19.6	19.4	3.8	4.4	5.0	5.1	2.7	2.6	6.3	6.3

**Table 3:** Normalized frequencies (%) of nucleotide changes in the chimpanzee genome and the categorized genomic regions after excluding CpG→TpG/CpA SNPs

Category	No. of SNPs	A→G	T→C	G→A	C→T	A→C	T→G	G→T	C→A	A→T	T→A	G→C	C→G
Genome	518,494	14.9	14.8	16.1	16.2	4.0	4.0	5.6	5.7	3.3	3.3	6.2	6.1
Intergenic regions	333,959	14.7	14.6	16.0	16.1	4.0	3.9	5.9	5.9	3.4	3.3	6.1	6.1
Genes	142,516	15.2	15.2	16.3	16.3	4.1	4.0	5.1	5.3	3.2	3.1	6.2	6.1
Introns	74,355	15.0	14.6	16.5	16.5	4.1	3.9	5.2	5.3	3.1	3.1	6.5	6.1
Exons	3074	17.5	16.8	17.3	17.9	3.2	3.3	4.1	4.2	2.3	2.0	5.6	5.8
CpG islands	5889	14.4	14.9	14.0	13.8	4.5	5.2	5.9	6.0	3.2	3.1	7.5	7.4

**Table 4:** Frequency decrease of G:C→A:T after removal of CpG→TpG/CpA SNPs

Category	GC content (%)	Frequency decrease of G:C→A:T (%)	
		
		Un-normalized	Normalized
Genome	40.0	8.8	9.7
Intergenic regions	39.4	8.2	9.2
Genes	41.0	9.7	10.4
Introns	39.7	8.8	9.7
Exons	**51.7**	**19.4**	**19.2**
CpG islands	62.0	12.1	11.2
			
Intergenic regions	<35 (31.4^a^)	5.7	7.1
	35–40 (37.3)	7.2	8.1
	40–45 (42.2)	9.0	9.6
	45–50 (47.2)	11.1	11.3
	**50–55 (52.2)**	**13.0**	**12.8**
	55–60 (57.2)	15.3	14.9
	≥ 60 (63.9)	18.2	17.4
	55–75 (60.2)^b^	16.6	16.0

^a^The average GC content (%) in the subcategory of intergenic regions.

^b^This GC content bin was included for the comparison with the CpG island category.
