# Supplementary material for: Directionality of point mutation and 5-methylcytosine deamination rates in the chimpanzee genome
Source: BMC Genomics. 2006 Dec 13;7:316. doi: 10.1186/1471-2164-7-316 (PMC1764022; doi:10.1186/1471-2164-7-316)
Supplement: Additional file 1 — Frequencies of nucleotide changes in intergenic regions. Supplementary Table S1 – Frequencies (%) of nucleotide changes in intergenic regions. [file 1471-2164-7-316-S1.pdf]

Table S1 - Frequencies (%) of nucleotide changes in intergenic regions

GC (%)[a]	No. of SNPs	A→G	T→C	G→A	C→T	A→C	T→G	G→T	C→A	A→T	T→A	G→C	C→G	u[d]	v[d]	*Observed* u / v	*Expected* u / v[e]
<35 (31.4[b])	69,874	18.6	18.6	13.9	13.8	5.1	5.0	4.2	4.0	4.8	4.5	3.8	3.7	35.9	47.3	1.66	2.19
35-40 (37.3)	60,205	17.2	17.2	16.1	15.9	4.5	4.4	4.3	4.3	3.7	3.9	4.2	4.4	40.5	43.3	1.57	1.68
40-45 (42.2)	42,540	15.5	15.6	18.2	18.6	4.0	4.1	4.2	4.1	3.1	3.1	4.6	4.9	45.1	39.2	1.58	1.37
45-50 (47.2)	24,821	13.3	13.2	21.3	21.2	3.6	3.6	4.5	4.2	2.7	2.5	4.9	5.0	51.3	33.6	1.71	1.12
50-55 (52.2)	14,196	11.4	11.3	23.6	23.7	3.2	2.7	4.4	4.7	2.4	2.2	5.3	5.1	56.4	28.6	1.81	0.92
55-60 (57.2)	7521	9.6	9.5	25.6	26.5	2.1	2.8	4.7	4.4	2.1	2.0	5.6	5.2	61.2	23.9	1.92	0.75
≥60 (63.9)	4500	6.8	8.0	28.1	27.8	2.2	1.8	4.7	5.4	1.5	1.3	6.2	6.1	66.0	18.9	1.97	0.57
55-75 (60.2)[c]	11,578	8.5	8.9	26. 7	27.1	2.1	2.4	4.7	4.8	1.8	1.7	5.8	5.6	63.2	21.9	1.91	0.66

[a]GC content in a non-repetitive intergenic region was obtained using a window size of 500 nucleotides.

[b]The average GC content (%) in the subcategory of intergenic regions.

[c]This GC content bin was included for the comparison with the CpG island category.

[d]u and v denote the rates of G/C→A/T and A/T→G/C, respectively

[e]Given no natural selection and independence of point mutation, Sueoka (1962) [1] proposed that the mean GC content at equilibrium (c) is expected to be $v / (u + v)$. This equation can be transformed to $u / v = (1-c) / c$. For SNP data,

$$u / v = \frac{N_u / f_{GC}}{N_v / (1 - f_{GC})} = \frac{N_u}{N_v} \times \frac{1 - f_{GC}}{f_{GC}}$$

where N_u and N_v are the numbers of G/C→A/T and A/T→G/C, respectively, and f_{GC} is the GC content.

References

1. Sueoka N: **On the genetic basis of variation and heterogeneity of DNA base composition.** *Proc Natl Acad Sci USA* 1962, **48:**582-592.
