# Supplementary material for: Directionality of point mutation and 5-methylcytosine deamination rates in the chimpanzee genome
Source: BMC Genomics. 2006 Dec 13;7:316. doi: 10.1186/1471-2164-7-316 (PMC1764022; doi:10.1186/1471-2164-7-316)

Figure S1 - Linear regression of the frequency of each type of nucleotide changes versus GC content in intergenic regions.

Intergenic regions were grouped into 7 GC-content bins. Solid lines denote nucleotide changes from G or C and dashed lines denote nucleotides from A or T.

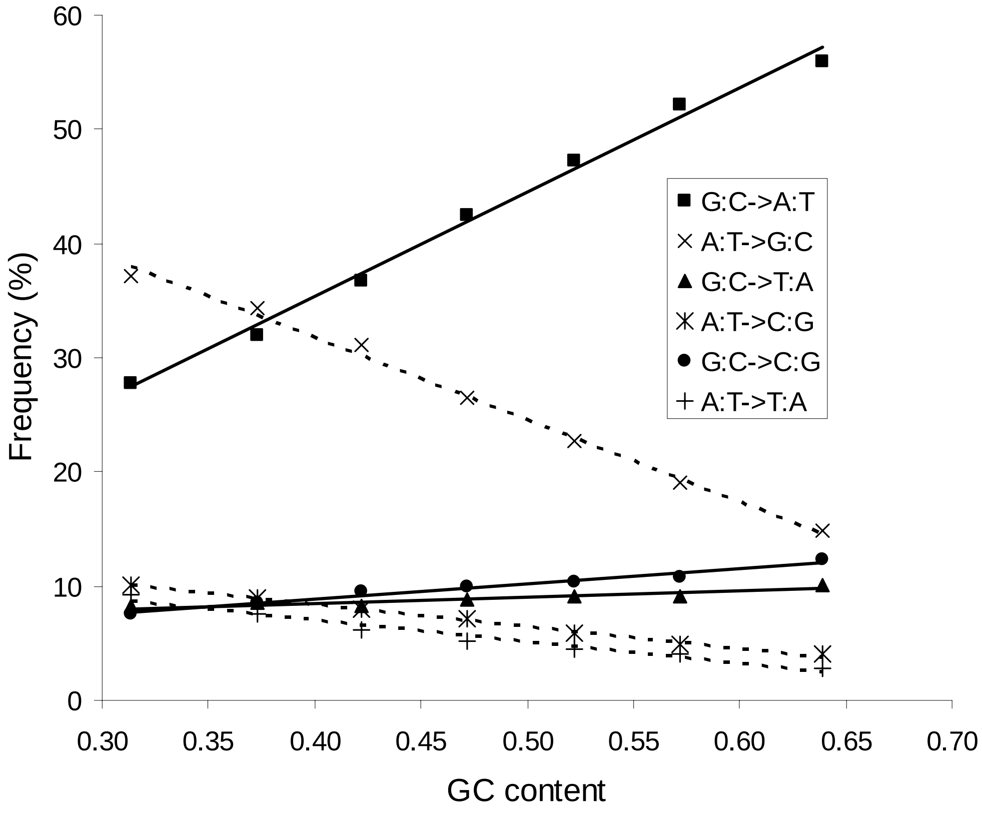

Supplement: Additional file 2 — Linear regression of the frequency of nucleotide changes versus GC content in intergenic regions. Supplementary Figure S1 – Linear regression of the frequency of each type of nucleotide changes versus GC content in intergenic regions. [file 1471-2164-7-316-S2.pdf]
