# Supplementary material for: Directionality of point mutation and 5-methylcytosine deamination rates in the chimpanzee genome
Source: BMC Genomics. 2006 Dec 13;7:316. doi: 10.1186/1471-2164-7-316 (PMC1764022; doi:10.1186/1471-2164-7-316)

**Figure S2 - Frequency difference of nucleotide changes between chimpanzees and humans in each genomic category.**

The frequency difference for each type of nucleotide changes was calculated by subtracting the frequency in the chimpanzee genome from that in the human genome.

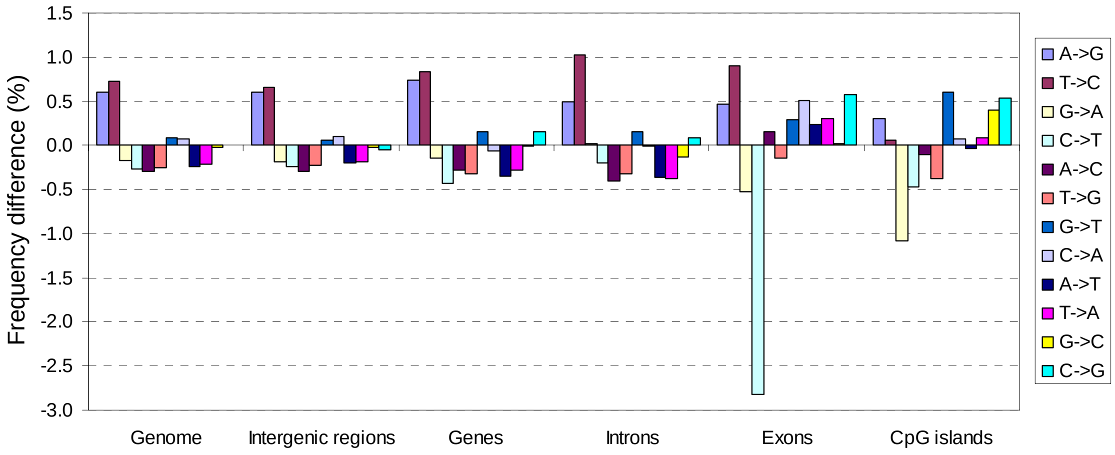

Supplement: Additional file 3 — Frequency difference of nucleotide changes between chimpanzees and humans in each genomic category. Supplementary Figure S2 – Frequency difference of nucleotide changes between chimpanzees and humans in each genomic category. [file 1471-2164-7-316-S3.pdf]
