# Supplementary material for: Directionality of point mutation and 5-methylcytosine deamination rates in the chimpanzee genome
Source: BMC Genomics. 2006 Dec 13;7:316. doi: 10.1186/1471-2164-7-316 (PMC1764022; doi:10.1186/1471-2164-7-316)

Figure S3 - Frequency difference of nucleotide changes for each pair of syntenic chimpanzee and human chromosomes.

The frequency difference for each type of nucleotide changes was calculated by subtracting the frequency in the chimpanzee genome from that in the human genome. The Y chromosome was not included due to the insufficient number of SNPs.

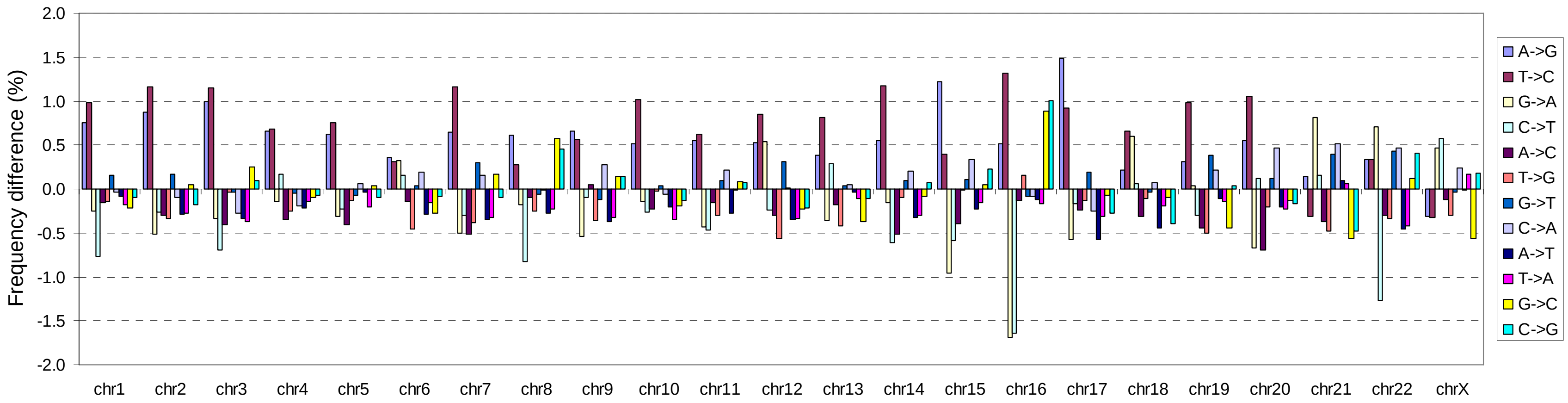

Supplement: Additional file 4 — Frequency difference of nucleotide changes for each pair of syntenic chimpanzee and human chromosomes. Supplementary Figure S3 – Frequency difference of nucleotide changes for each pair of syntenic chimpanzee and human chromosomes. [file 1471-2164-7-316-S4.pdf]
